# Combination Therapy With Fecal Microbiota Transplantation and Vedolizumab for Induction of Remission in Ulcerative Colitis: An Open-Label Pilot Study

**DOI:** 10.1093/ibd/izaf284

**Published:** 2025-12-10

**Authors:** Saad Syed, Paul Moayyedi, Dina Kao, Jaiminkumar Patel, John K Marshall, Michael Surette, Neeraj Narula

**Affiliations:** Department of Medicine and Farncombe Family Digestive Health Research Institute, McMaster University, Hamilton, ON, Canada; Department of Medicine and Farncombe Family Digestive Health Research Institute, McMaster University, Hamilton, ON, Canada; Department of Medicine, University of Alberta, Edmonton, AB, Canada; Department of Medicine and Farncombe Family Digestive Health Research Institute, McMaster University, Hamilton, ON, Canada; Department of Medicine and Farncombe Family Digestive Health Research Institute, McMaster University, Hamilton, ON, Canada; Department of Medicine and Farncombe Family Digestive Health Research Institute, McMaster University, Hamilton, ON, Canada; Department of Medicine and Farncombe Family Digestive Health Research Institute, McMaster University, Hamilton, ON, Canada

**Keywords:** Ulcerative Colitis, Vedolizumab, Combination Therapy, Fecal Transplant, Microbiome

## Introduction

The etiology of ulcerative colitis (UC) is classically described as an interaction among genetic, environmental, and microbiological factors leading to a dysregulated immunologic response. Treatment is largely targeted at dampening this immune response. Previous attempts to address microbiological factors with probiotics and antibiotics have largely failed to demonstrate benefit. Over the last decade, fecal microbiota transplantation (FMT) has shown promise in several randomized controlled trials for inducing remission in UC.^1^

Minimal data exists combining advanced therapy with FMT in UC—one recent case study highlighted this potential approach—and there is significant interest in combining therapies in UC.^2^ At the same time, there is concern for adverse effects of FMT in immune-compromised patients.^3^ Here, we present a pilot study to assess the safety, feasibility, and efficacy of using FMT combined with vedolizumab as induction therapy in participants with UC who have previously failed an advanced medical therapy.

## Methods

This is a prospective open label pilot study involving a single center (McMaster University Medical Centre) recruiting participants from Hamilton, Ontario, Canada, using FMT via enema once weekly for 6 weeks in combination with vedolizumab for induction of remission of moderately to severely active UC in patients who previously have failed at least 1 other advanced therapy. A complete list of the primary and secondary outcomes for this study is available on ClinicalTrials.gov (NCT04231110). The study was approved by the Hamilton Integrated Research Ethics Board and Health Canada (control #239034) and was conducted utilizing Good Clinical Practice principles.

The primary goal of the study was to assess feasibility of recruitment and feasibility for participants to complete FMT intervention weekly while receiving vedolizumab. Exploratory outcomes included clinical response as defined by total Mayo score ≤4 with at least a 2-point drop from baseline and with endoscopic subscore ≤1), adverse events, and stool microbial analyses.

FMT products were manufactured from a single screened donor registered with the Edmonton FMT program (University of Alberta). Donor qualifying and testing processes and FMT manufacturing and quarantine protocols have been described previously.^4^ Briefly, 100 g of donor stool was mixed with 200 cm^3^ of water, filtered through a stomacher bag, and then divided into approximately 13 doses of 15 cm^3^ enemas and stored at −80 °C until use.

Eligible participants were adults (≥18 years of age) with clinically active UC (total Mayo score ≥5) who were initiating vedolizumab. Participants had to have failed at least 1 prior advanced therapy, with no restrictions on which advanced therapy failures they may have experienced. Participants were excluded if they had previously been exposed to vedolizumab or other anti-integrin therapy, were participating in another clinical trial, were unable to give consent, had concomitant *Clostridium difficile* infection, had lactose intolerance, had severe comorbid medical illness, were pregnant, or had a recent change in UC-related medical therapies (i.e., increasing corticosteroids, antibiotics, new probiotics, or change in advanced therapy dosing) in the prior 28 days.

After completing informed consent, participants underwent baseline assessments with flexible sigmoidoscopy, clinical interview, blood and stool tests. Participants provided a stool sample within 2 weeks of the initial study visit for baseline microbiome assessment and at the final evaluation visit at 7 weeks, which also included a second assessment with flexible sigmoidoscopy. Participants were otherwise assessed weekly at the time of FMT administration for the patient-reported outcomes portion of the Mayo score as well as for any adverse effects clinically for 30 minutes after administration. Clinical outcomes were reviewed descriptively.

DNA extraction, sequencing, and microbial composition analysis was undertaken as previously described.^5^ All sequences were clustered into operational taxonomic units (OTUs) at 99% similarity. All analyses were conducted in RStudio (version 2023.12.1 + 402) with R (version 4.3.2; R Foundation for Statistical computing) using the microViz, tidyverse, phyloseq, vegan, ggplot2, and UpSet packages for analysis and visualization.

## Results

The study was initiated in February 2020 with the plan to recruit 10 active UC patients within 1 year. Initial delays occurred due to the COVID-19 pandemic, which limited use of FMT for several months. The trial was terminated after 7 participants were recruited over 4 years.

All participants received FMT weekly for 6 weeks. Adverse events were not noted in any participants, and all enrolled participants completed the trial.

Participant baseline demographics and characteristics are listed in Table 1. The median age of participants was 26 years of age (range 18-65 years). Four participants had previously failed 1 advanced therapy and 3 had failed 2 or more advanced therapies.

**Table 1.** Summary of patients and their outcomes.

	Case	Sex	Age at diagnosis (y)	Age (y)	Disease phenotype	Previous agents	Concomitant agents	Ulcerative colitis activity			Adverse events
								Marker	Baseline	End	
**Responders**	1	F	27	34	Proctitis	Adalimumab Prednisone	None	CRP, mg/L	<5	1.5	None
								Fecal calprotectin, µg/g	63.9	N/A	
								Mayo score	7	3	
	2	M	24	26	Pancolitis	Azathioprine Infliximab	None	CRP, mg/L	0.6	15.6	None
								Fecal calprotectin, µg/g	N/A	126.1	
								Mayo score	8	3	
**Nonresponders**	3	M	54	65	Left sided	5-ASA Tofacitinib	5-ASA	CRP, mg/L	16.7	47.5	None
								Fecal calprotectin, µg/g	141.9	1392.2	
								Mayo score	8	7	
	4	F	23	24	Proctitis	5-ASA Adalimumab Azathioprine	None	CRP, mg/L	0.7	<5	None
								Fecal calprotectin, µg/g	269.3	53.6	
								Mayo score	7	11	
	5	M	29	35	Left sided	Adalimumab Infliximab Methotrexate Probiotics	Probiotics	CRP, mg/L	37.4	80.5	None
								Fecal calprotectin, µg/g	109.3	658.6	
								Mayo score	7	11	
	6	M	17	18	Left-sided	5-ASA Probiotics Ustekinumab	Probiotics	CRP, mg/L	12.6	18.4	None
								Fecal calprotectin, µg/g	1474	1558	
								Mayo score	10	13	
	7	M	13	23	Pancolitis	5-ASA Adalimumab Antibiotics Azathioprine Infliximab Prednisone Probiotics Ustekinumab	Probiotics	CRP, mg/L	53.3	18.9	None
								Fecal calprotectin, µg/g	3139	1533	
								Mayo score	9	14	

Abbreviations: F, female; M, male; N/A, not applicable; 5-ASA, 5-aminosalicylic acid.

At study completion, 2 of the 7 participants had clinical response as each had a total Mayo score of 3, with both achieving endoscopic remission with endoscopic Mayo scores of 0. Both responders had 1 prior advanced therapy exposure and low inflammatory markers. One of the 2 responders (case 2) had resolution of rectal bleeding and normalization of stool frequency after study week 1 enema and initial vedolizumab transfusion, whereas the second responder (case 1) did not have any change in the patient reported outcomes of rectal bleeding or stool frequency throughout the study but attained endoscopic remission.

**Figure 1.**
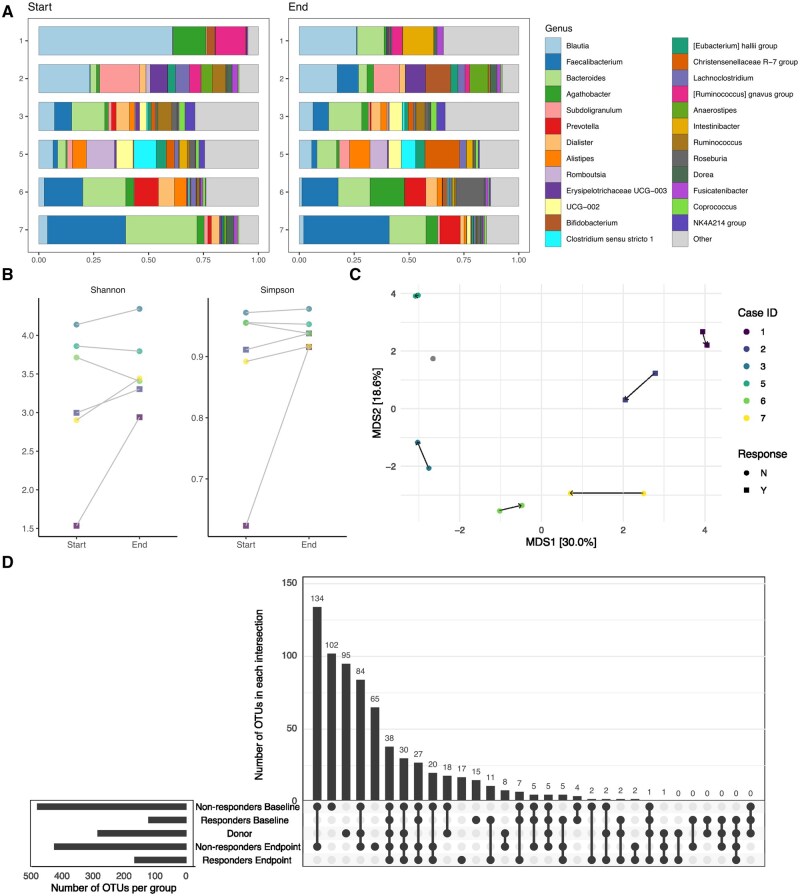
Fecal microbiome analysis by participant and responder status, before and after fecal microbial transfer. (A) Relative abundance taxonomic summaries by first sample and endpoint sample and ordered by participant for the 6 participants who provided samples. Top 25 genera in the data were colored and are identified in the legend. All other genera are in “other.” (B) Participants’ fecal microbial α-diversity assessed by Shannon and Simpson indices at baseline (n = 6), at the start and end of the study. Each dot represents the value of the diversity metric from each sample. The dots are colored by case ID. Response to therapy is indicated by shape. (C) Participants’ fecal microbial β-diversity assessed by Aitchison distance and visualized using a principal coordinate analysis plot. Each dot represents a sample and samples are colored by case ID. Response to therapy is indicated by shape. Arrows connect the first sample to the endpoint sample for each participant. Percent variation explained by each axis is noted in square brackets. (D) UpSet plot comparing operational taxonomic units (OTUs) present in each group as indicated by the connected dots, and separated into categories of donor (n = 1), responders (n = 2), and nonresponders (n = 4). The y-axis shows the number of OTUs common between the groups identified by the connected dots. The bar graph beside the x-axis shows the total number of OTUs detected in each group.

One participant (case 4) did not provide baseline or endpoint stool samples and was excluded from microbiome analyses. After removing nonbacterial reads, 516 797 reads remained (median of 33 404 [range: 22 114-69 496] per sample) and 837 OTUs remained (median of 151 [range 55-284] per sample). Fecal microbiome composition was assessed using taxa bar summaries (Figure 1A). Both responders were noted to have an increase in α-diversity by Shannon and Simpson diversity at the end of the study, whereas only 2 of the 4 nonresponders did (Figure 1B). β-Diversity demonstrated clustering by case, as well as by responders vs nonresponders (Figure 1C). No clustering was noted between beginning and end point samples across cases. Using an UpSet plot to evaluate the number of OTUs shared between donor and recipients at the study endpoint, we found that 9 total OTUs were shared in the donor sample and in at least 1 of the recipients and were not present in any baseline samples (Figure 1D). These were felt to be transferred OTUs from the donor to recipients, although, notably, no OTUs were solely shared by the donor with responders at endpoint.

## Discussion

This study sought to assess the safety, feasibility, and efficacy of combining vedolizumab with FMT for the induction of remission of moderately to severely active UC. We found 2 of 7 of participants to have clinical response after 6 weekly FMT enemas, with no adverse events associated with combining these therapies in our study.

Our study failed to recruit to our target enrollment. Likely, this relates to evolving prescribing patterns in the care of patients with UC. A post-hoc analysis of the GEMINI-1 trial (Vedolizumab as Induction and Maintenance Therapy for Ulcerative Colitis) has suggested reduced efficacy of vedolizumab in patients who have failed other advanced therapies.^6^ Conversely, the VARSITY trial (Vedolizumab versus Adalimumab for Moderate-to-Severe Ulcerative Colitis) demonstrated superiority of vedolizumab over adalimumab for the induction of remission in UC, but the study population was limited to mostly biologic-naïve participants.^7^ These studies likely led to vedolizumab being preferentially positioned as a first-line advanced therapy, and therefore reduced the number of patients eligible for recruitment.

Regarding efficacy, we did observe clinical response in 2 of our 7 patients, each of whom had total Mayo scores of 3 and complete endoscopic remission at study endpoint. Future studies with a control arm (monotherapy vedolizumab or FMT) are needed to better characterize any synergistic benefits for efficacy in the induction of remission and determine microbiome effects of FMT vs from improving inflammation from vedolizumab.

Regarding microbiome markers, by the study endpoint, both responders demonstrated improvement in their α-diversity. Interestingly, both patients also clustered together with respect to their β-diversity, which may support the role of the recipient microbiome and its influence on response to FMT.^8^ Additionally, only a handful of OTUs were noted to be transferred between donor and recipients, and none were transferred to only clinical responders. Some studies have found an association between higher donor microbiota similarity and clinical response following FMT, but others have not.^9^^,^^10^ The relationship between donor microbiota similarity and clinical response remains unclear and may also relate to detection limits associated with different sequencing approaches.^5^^,^^9^

Our study has several limitations. The number of participants is small. FMT, including weekly dosing and duration in our study, may limit recruitment due to time and psychological barriers. Our regimen conversely may not be sufficient to induce remission in this patient population. A single donor was used to minimize treatment heterogeneity; such an approach may potentially contribute to lower efficacy if this donor is not the “right match” for a recipient. The results may not be applicable to those starting a different biologic than vedolizumab. Our methodology cannot provide strain-level information, which has been shown to correlate with treatment response.^5^

Further studies should continue to explore where to position FMT in the IBD treatment algorithm, and if FMT as an adjunct to advanced therapies can improve treatment efficacy or reduce adverse events or recurrence of flares. As microbial therapy evolves from FMT to defined bacterial consortia, identification of specific strains that may synergize with unique mechanisms of immune modulation may provide greater safety and efficacy.

## Data Availability

The datasets and scripts underlying this study will be shared on reasonable request to the corresponding author.
